# Formation and Identification of Lignin–Carbohydrate Complexes in Pre-hydrolysis Liquors

**DOI:** 10.1021/acs.biomac.3c00053

**Published:** 2023-06-02

**Authors:** Nianjie Feng, Shaowen She, Fei Tang, Xiangdong Zhao, Jingqian Chen, Peng Wang, Qian Wu, Orlando J. Rojas

**Affiliations:** † Key Laboratory of Fermentation Engineering (Ministry of Education), Hubei Key Laboratory of Industrial Microbiology, National “111” Center for Cellular Regulation and Molecular Pharmaceutics, Hubei Research Center of Food Fermentation Engineering and Technology, Hubei University of Technology, Wuhan 430068, China; ‡ Bioproducts Institute, Department of Chemical & Biological Engineering, Department of Chemistry, and Department of Wood Science, The University of British Columbia, 2360 East Mall, Vancouver BC V6T 1Z3, Canada; § Department of Bioproducts and Biosystems, School of Chemical Engineering, Aalto University, Vuorimiehentie 1, FI-00076 Espoo, Finland

## Abstract

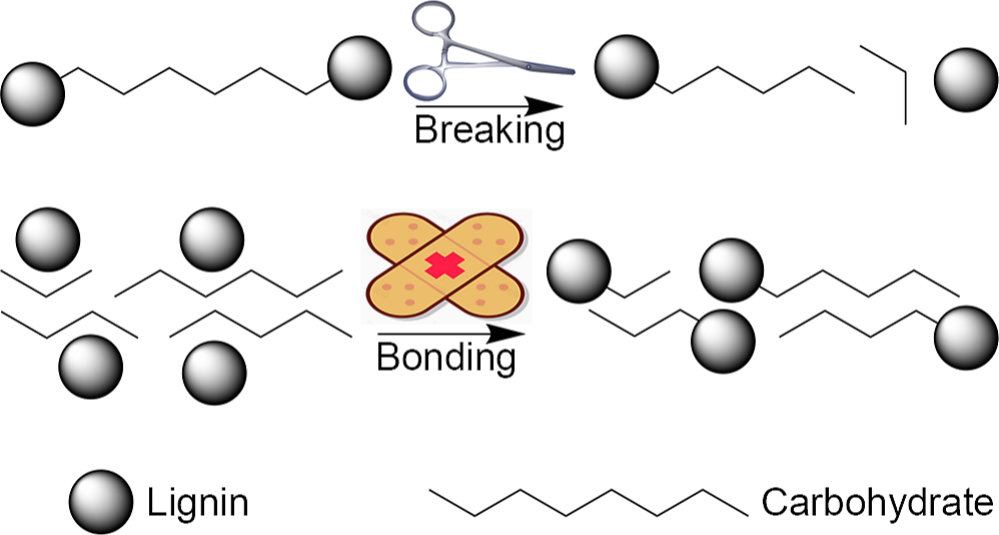

The lignin–carbohydrate complexes (LCCs) typically present in the liquors produced in the pre-hydrolysis of biomass cause severe difficulties in downstream fractionation. To address this issue, a series of LCC samples were accessed from solutions obtained from the pre-hydrolysis of extractive-free pine wood meal (H-LCC) and compared with LCC obtained from the corresponding residues (B-LCC). Chromatographic and spectroscopic techniques revealed that 8.2% of the lignins were degraded at 160 °C, resulting from the breakage of β-O-4′ linkages during pre-hydrolysis. Meanwhile, (reactive) hemicelluloses were mainly removed from the fibers’ cell walls. Some hemicelluloses in the pre-hydrolysis liquor, such as glucomannans, were associated with degraded lignin fragments via ether and ester bonds. However, the newly formed LCCs were pH-labile and underwent rapid hydrolysis. Overall, we reveal details about LCC formation and degradation during pre-hydrolysis at given temperatures, critically important in efforts to improve biomass processing and valorization.

## 1 Introduction

Masson pine is a softwood with hemicelluloses mainly consisting of O-acetyl-galactoglucomannans (GGM) and arabino-4-O-methylglucuronoxylans (AGX). The lignin present guaiacyl-type β-O-4′ structures and show a relative abundance of C–C linkages (5–5′ and β-5′). The hemicelluloses and lignin are closely connected as is typically the case of other wood fibers, forming a nanoscale network structure.^1−3^ The major barriers for lignocellulosic biomass conversion include wood’s heterogeneous chemistry and structure.^4^ Hence, pre-hydrolysis has been used to separate the chemical components in plant cell walls, with water used as reaction medium.^5^ The process uses temperatures in the range between 150 and 220 °C and pressures at saturation or higher levels. As a result, the majority of hemicelluloses are dissolved from the cell wall into the pre-hydrolysis liquor, and cellulose is enriched in the pre-hydrolysis residue, which can be used for the production of dissolving-grade pulps.^6^

Recent studies have focused on the isolation and conversion of hemicelluloses present in the pre-hydrolysis liquors.^7,8^ However, related streams often contain given amounts of lignins.^9^ In this context, the hydrolysis of acetyl groups is of interest since acetic acid is produced, promoting lignin’s bond breaking during pre-hydrolysis, facilitating dissolution.^10,11^ Moreover, it is generally accepted that some co-dissolved lignins are gradually deposited in the pre-hydrolysis liquor and “connect” with hemicelluloses.

Back in 1866, Erdmann hypothesized that the presence of covalent bonds between lignins and carbohydrates (“glycolignose”) is the main reason for the difficulties observed in separating the two components, nowadays clearly shown to include benzyl ether, benzyl ester, and phenyl glucoside linkages.^12,13^ The compounds involved in these linkages are the lignin–carbohydrate complexes (LCC), which are found in pre-hydrolysis liquors.^14^

The occurrence of lignin–carbohydrate bonds creates significant problems in the selective isolation of lignins and carbohydrates.^15^ This is also one of the key challenges limiting the high-value utilization of pre-hydrolysis liquors. Hence, there is a need to gain a better understanding of the structure of LCCs and their formation mechanism. This is a topic addressed in this study, where LCCs were extracted from the liquors, along with respective residues, obtained by the pre-hydrolysis of Masson pine. We studied the changes in the chemical structure, under different reaction temperatures, by means of high-performance liquid chromatography (HPLC), Fourier transform infrared (FTIR) spectroscopy, ultraviolet–visible (UV–vis) spectroscopy, gel permeation chromatography (GPC), two-dimensional heteronuclear single-quantum coherence nuclear magnetic resonance (2D HSQC NMR) spectroscopy, and ^13^C nuclear magnetic resonance (^13^C NMR) spectroscopy.

## 2 Materials and Methods

### 2.1 Materials

Masson pine (*Pinus massoniana*) chips were collected from Jiangxi Academy of Forestry, China. The chips were ground (18–42 mm) using a laboratory mill. The chips were extracted (Soxhlet) using a refluxing mixture of benzene and ethanol (v/v, 2/1) for 12 h. The extractive-free pine meal was washed and air-dried for subsequent pre-hydrolysis. KBr, (Dimethyl sulfoxide)-*d*_6_ (DMSO-*d*_6_), and chromium(III) acetylacetonate were purchased from Sigma-Aldrich (St. Louis, MO). All other chemicals and reagents used were purchased from Sinopharm Chemical Reagent Co., Ltd (Beijing, China).

### 2.2 Pre-hydrolysis

The extractive-free pine meal (40 g, oven dry) was extracted with 400 g deionized water in 1-L reactors (Parr 4530, USA), equipped with a temperature controller, a pressure gauge, and a mechanical stirrer. Pre-hydrolysis was performed at temperatures from 140 to 160 °C for 120 min based on our previous report.^16^

### 2.3 LCC Isolation from the Pre-hydrolysis Liquor

The LCC isolated from pre-hydrolysis liquors, herein referred to as H-LCC, were obtained as previously described.^16^ Shortly, LCC-containing samples were concentrated, dried, and dissolved in dimethyl formamide (DMF). Then, a continuous purification was performed by using 1,2-dichloroethane/ethanol (v/v, 2/1), diethyl ether, acetone/acetic acid (v/v, 99/1), diethyl ether, and petroleum ether. The obtained H-LCC samples included H-LCC_140_, H-LCC_150_, and H-LCC_160_ according to the respective treatment temperature. The solid content of the pre-hydrolysis liquor was determined from the oven-dried mass determined at 105 °C. The yield was calculated as H-LCC weight percentage based on the solid content.

### 2.4 LCC Isolation from Pre-hydrolysis Residues

LCC from the pre-hydrolysis residues, herein referred to as B-LCC, were obtained from the residues that were dried and ground for 72 h in a vibration ball mill under water cooling. The B-LCC samples were extracted and purified using the Björkman method,^17^ yielding B-LCC_140_, B-LCC_150_, and B-LCC_160_, according to the respective treatment temperature. The LCC directly obtained from the extractive-free pine meal, using the same conditions, was used as a control and named as B-LCC_M_. The yield was calculated as B-LCC weight percentage in relation to the precursor (non-milled) materials.

### 2.5 LCC Chemical Analysis

LCC chemical composition analysis was conducted by hydrolysis with a two-step sulfuric acid.^18^ The solid obtained after the acid hydrolysis was Klason lignin. The filtrates were used for acid-soluble lignin and sugar determination. The acid-soluble lignin was measured using a UV–vis spectrophotometer (HITACHI U-3900, Japan). The sum of Klason lignin and acid-soluble lignin yielded the total lignin content. Sugars were determined by HPLC (Shimadzu, COT-20A, Japan) equipped with a refractive index detector (Shimadzu) on an Aminex HPX-87P column (Bio-Rad, Hercules, USA), with water as the eluent.

### 2.6 LCC Molecular Weight

The molecular weights of the LCC samples were determined using gel permeation chromatography (GPC, Agilent, USA) equipped with a PL-gel 10 mm mixed-B 7.5 mm i.d. column and a refractive index detector. Five milligrams of LCC was dissolved in 2 mL of tetrahydrofuran (THF). Monodisperse polystyrene was used as the standard.

### 2.7 FTIR and UV–Vis Analyses

LCCs were analyzed by FTIR (Thermo Fisher 6700, USA) according to the published paper.^3^ Two milligrams of the sample was milled, dispersed in spectroscopic-grade KBr, and subsequently pressed into disks. A total of 64 scans with a 2 cm^–1^ resolution were signal-averaged. The wavenumber range scanned was 4000–500 cm^–1^.

The LCC samples were also characterized by a UV–vis spectrophotometer (HITACHI U-3900, Japan). 5 mg of the sample was dissolved in 10 mL of 95% (v/v) dioxane/water and then diluted 10 times with 50% (v/v) dioxane/water.^19^ The absorbance between 260 and 420 nm was measured.

### 2.8 LCC Spectroscopy Analyses

2D HSQC NMR and ^13^C NMR spectroscopy analyses of LCC samples were carried out using a Bruker AVANCE 600 MHz spectrometer. For the 2D-HSQC NMR experiments, 40 mg of LCC was dissolved in 0.5 mL of DMSO-*d*_6_. The contribution of the various substructures and linkages in LCC preparations were calculated from the NMR spectra according to the work of Wen et al. (2013) and Huang et al. (2016).^20,21^ For the quantitative ^13^C NMR experiments, 100 mg of LCC was dissolved in 0.5 mL of DMSO-*d*_6_ and 40 μL of chromium(III) acetylacetonate (0.01 M).

### 2.9 Statistical Analyses

Data are reported as mean ± standard deviation (S.D.). Significant differences were determined using one-way ANOVA, followed by Tukey’s post-hoc analysis. *P* < 0.05 was considered significant. All statistical analyses were performed using SPSS 20.0, and the graphs were generated using Origin 8.0.

## 3 Results and Discussion

### 3.1 Degradation of Masson Pine

**Figure 1:**
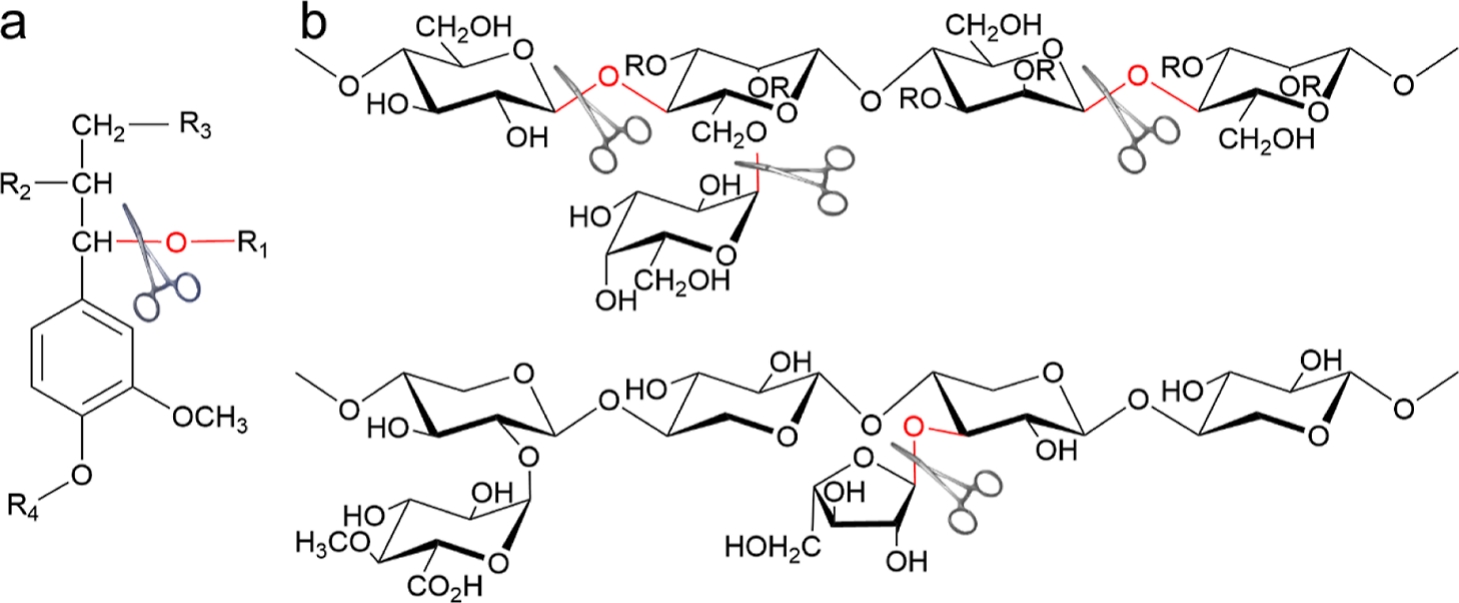
Possible reactions of lignins (a) and hemicelluloses (b) during the pre-hydrolysis process. The scissors indicate the breaking of chemical bonds.

The changes in the chemical composition of Masson pine during pre-hydrolysis are summarized in Table 1. The α-ether groups of lignin were cleaved readily under the simultaneous formation of benzylium ions during pre-hydrolysis (Figure 1a). The cleavage of open α-aryl ether bonds represented a noteworthy fragmentation. Due to relatively few α-ether groups present in the (softwood) lignins, only 7.8% of lignin was degraded at 160 °C (Table S2). Meanwhile, due to the cleavage of glycosidic linkages under acidic hydrolysis, polysaccharide depolymerization occurred during pre-hydrolysis. Moreover, hemicelluloses were attacked more readily than celluloses due to their branched, amorphous structure and their relatively low degree of polymerization.^22^ With the increased severity of hydrolysis, the depolymerized hemicellulose fragments were dissolved in the pre-hydrolysis liquor. Finally, cellulose depolymerization was minimal and had no significant contribution to the yield losses.

**Table 1:** Chemical Compositions of the Residues and Extracts^a^

chemical composition	raw material	residues			extracts		
		140	150	160	140	150	160
yield (%)		86.1 ± 0.7	84.1 ± 0.9	80.6 ± 0.4			
lignin (%)	29.8 ± 0.5	33.0 ± 0.3	33.4 ± 0.3	34.1 ± 0.6	5.2 ± 0.1	5.4 ± 0.1	5.7 ± 0.1
glucose (%)	43.7 ± 0.7	47.0 ± 0.6	47.2 ± 0.7	47.7 ± 0.4	12.0 ± 0.2	12.2 ± 0.2	12.9 ± 0.1
xylose (%)	5.3 ± 0.2	2.3 ± 0.2	2.1 ± 0.1	1.9 ± 0.1	11.5 ± 0.1	10.3 ± 0.1	8.8 ± 0.1
galactose (%)	2.9 ± 0.1	1.0 ± 0.0	0.7 ± 0.1	0.5 ± 0.1	6.9 ± 0.1	6.6 ± 0.2	5.7 ± 0.1
arabinose (%)	0.7 ± 0.1	0.1 ± 0.1	N.D	N.D	2.2 ± 0.0	1.9 ± 0.1	1.6 ± 0.1
mannose (%)	16.2 ± 0.3	6.2 ± 0.2	5.5 ± 0.2	3.7 ± 0.2	37.3 ± 0.4	33.4 ± 0.7	30.9 ± 0.5
4-O-methyl-glucuronic acid (%)	1.4 ± 0.3	1.5 ± 0.1	1.5 ± 0.2	1.5 ± 0.2	1.2 ± 0.1	1.2 ± 0.1	1.3 ± 0.2
solid content (mg/mL)					11.3 ± 0.2	13.5 ± 0.3	16.7 ± 0.1

a Mass percentage is reference to the respective samples (yield relative to o.d. biomass). The headings used (140, 150, and 160) represent the samples from the different pre-hydrolysis temperatures. Data are mean ± S.D. N.D. indicates “not detected”. The absolute mass and chemical compositions are presented in Table S1 and S2, respectively.

Figure 1b shows the changes that occurred in the main hemicelluloses, which included GGM. Galactose was usually regarded as side chains in GGM and hydrolyzed together with a glycosidic bond. AGX, the other main hemicellulose constituent of softwoods, was degraded to glucuronoxylan. Because the furanosidic linkages of the arabinose units are extremely labile toward protons, it is likely that they were cleaved already at the early stages of pre-hydrolysis.^23,24^ The glycuronide bonds of 4-O-methyl-glucuronic acid were stable toward acid, contrary to the glycosidic bonds.^25^

The chemical compositions of extracts are listed in Table 1. Because only a small amount of lignin was degraded, its content in the extract increased slightly, from 5.2 to 5.7%. The change of glucose content was similar, with a small increase. The content of mannose in the extract reached up to 37.3% (140 °C). Hemicelluloses are relatively prone to hydrolysis by high temperature and acids. Except 4-O-methyl-glucuronic acid, the hemicellulosic saccharide content presented a sharp reduction with hydrolysis temperature (Table 1).

### 3.2 LCC Composition

The chemical composition of H-LCC is included in Table 2. As the reaction temperature increased, the yield of H-LCC first increased and then decreased, with a peak at 150 °C. The increase in yield indicated that new LCCs were formed. However, it remained ambiguous whether the LCCs were from dissolution or bonding. The lignin content kept falling during pre-hydrolysis. On the contrary, the content of glucose and mannose increased with hydrolysis temperature. Mannose was the main component in the H-LCC samples leading to the conclusion that H-LCCs from the Masson pine pre-hydrolysis liquor may be present in the form of lignin–glucomannan complexes. In addition, the increase in 4-O-methyl-glucuronic acid content seemed to indicate the presence of some lignin–glucuronoxylan complexes.

**Table 2:** Chemical Composition of H-LCC and B-LCC Obtained after Pre-hydrolysis at 140, 150, and 160 °C for 120 min^a^

chemical compositions	H-LCCs			B-LCCs			
	140	150	160	M	140	150	160
yield (%)	7.7 ± 0.2	14.0 ± 0.4	13.4 ± 0.4	13.5 ± 0.4	8.1 ± 0.2	4.9 ± 0.1	2.3 ± 0.2
lignin (%)	6.7 ± 0.1	5.2 ± 0.1	5.0 ± 0.1	29.6 ± 0.5	39.9 ± 1.3	46.5 ± 1.3	51.1 ± 1.4
glucose (%)	16.3 ± 0.2	20.4 ± 0.2	23.0 ± 0.3	12.7 ± 0.2	13.5 ± 0.1	13.5 ± 0.3	13.8 ± 0.1
xylose (%)	15.6 ± 0.2	12.0 ± 0.2	9.5 ± 0.3	7.5 ± 0.1	6.8 ± 0.2	4.7 ± 0.1	2.6 ± 0.1
galactose (%)	19.6 ± 0.1	17.5 ± 0.7	14.3 ± 0.2	19.8 ± 0.9	10.0 ± 0.1	8.1 ± 0.3	7.9 ± 0.2
arabinose (%)	0.7 ± 0.1	0.5 ± 0.1	0.4 ± 0.0	0.8 ± 0.2	0.3 ± 0.1	0.2 ± 0.0	0.2 ± 0.1
mannose (%)	39.5 ± 0.7	42.7 ± 1.2	45.7 ± 0.8	28.1 ± 1.1	27.8 ± 0.5	25.3 ± 0.7	22.5 ± 0.8
4-O-methyl-glucuronic acid (%)	1.6 ± 0.2	1.7 ± 0.1	2.1 ± 0.3	1.5 ± 0.1	1.7 ± 0.2	1.7 ± 0.1	1.9 ± 0.3

a The mass percentage is related to the respective samples (relative to o.d. biomass). The headings used (140, 150, and 160) represent the samples from the different pre-hydrolysis temperatures. The “*M*” heading is used to indicate the LCCs extracted from the raw material. Data are mean ± S.D. The absolute mass of the chemical compositions is presented in Table S3.

It is well known that LCCs are prone to deconstruction under both acidic and alkaline conditions, depending on whether the linkages between lignins and carbohydrates involve ether or ester bonds.^26^ The yield of B-LCC_160_ decreased to only 2.3%, suggesting that LCCs in the plant cell wall were deconstructed during pre-hydrolysis. With increasing temperature, the lignin content in B-LCC increased, while the content of most carbohydrates decreased, similar to the results of degradation of Masson pine residues (Table 1). The fact that the trends for galactose and mannose contents in H-LCCs were different meant that GGM was not as a whole bonded with lignin. Another key point is that the trends for galactose content in H-LCCs were opposite to that in B-LCCs. Hence, it is reasonable to assume that some LCCs were not directly dissolved from the cell wall during the pre-hydrolysis treatment.

### 3.3 LCC Molecular Weight Distribution

**Figure 2:**
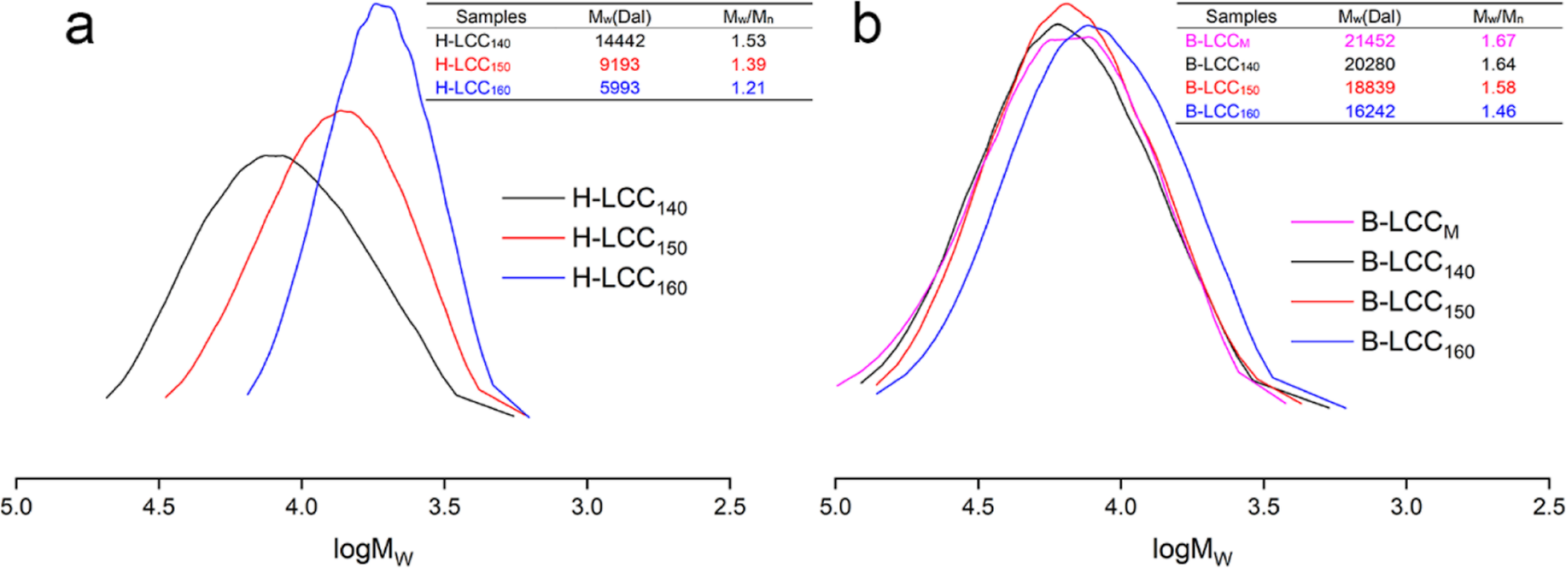
Molecular mass distribution of H-LCC (a) and B-LCC (b) obtained after pre-hydrolysis at 140, 150, and 160 °C for 120 min. The inset tables correspond to the weight average molecular weight (*M*_w_) and polydispersity index (*M*_w_/*M*_n_).

The molecular weight of H-LCC is shown in Figure 2a. The weight-average molecular weight (*M*_w_) of H-LCC samples drastically decreased with increasing temperature, from 14,442 to 5993 Da. Meanwhile, the polydispersity (*M*_w_/*M*_n_) decreased with the increased temperature. Combined with the data of *M*_w_ and *M*_w_/*M*_n_, it is apparent that H-LCC depolymerization occurred, mainly as a consequence of carbohydrate hydrolysis. It is possible that the formation and depolymerization of LCC occurred simultaneously during pre-hydrolysis. Based on the change of lignin-to-carbohydrate ratio in H-LCC (Table 2), one can speculate that the formed species were carbohydrate-enriched LCC (hydrophilic), while the depolymerized LCC tended to be lignin-enriched LCC (hydrophobic).

The *M*_w_ and *M*_w_/*M*_n_ of B-LCC were reduced (Figure 2b) with the increased hydrolysis temperature. Glycosidic bonds in the molecular backbone structure were broken, according to the HPLC results. Compared with the B-LCC, lower *M*_w_ and *M*_w_/*M*_n_ were found in H-LCC. This indicates that H-LCC had undergone more severe hydrolysis in the bulk solution.^27^ Up to 160 °C, however, the absolute mass of H-LCC_160_ was greater than that of B-LCC_160_ (Table S3). Therefore, it is reasonable to propose that H-LCC could not only come from the LCC dissolved from Masson pine.

### 3.4 FTIR and UV–Vis of LCC

**Figure 3:**
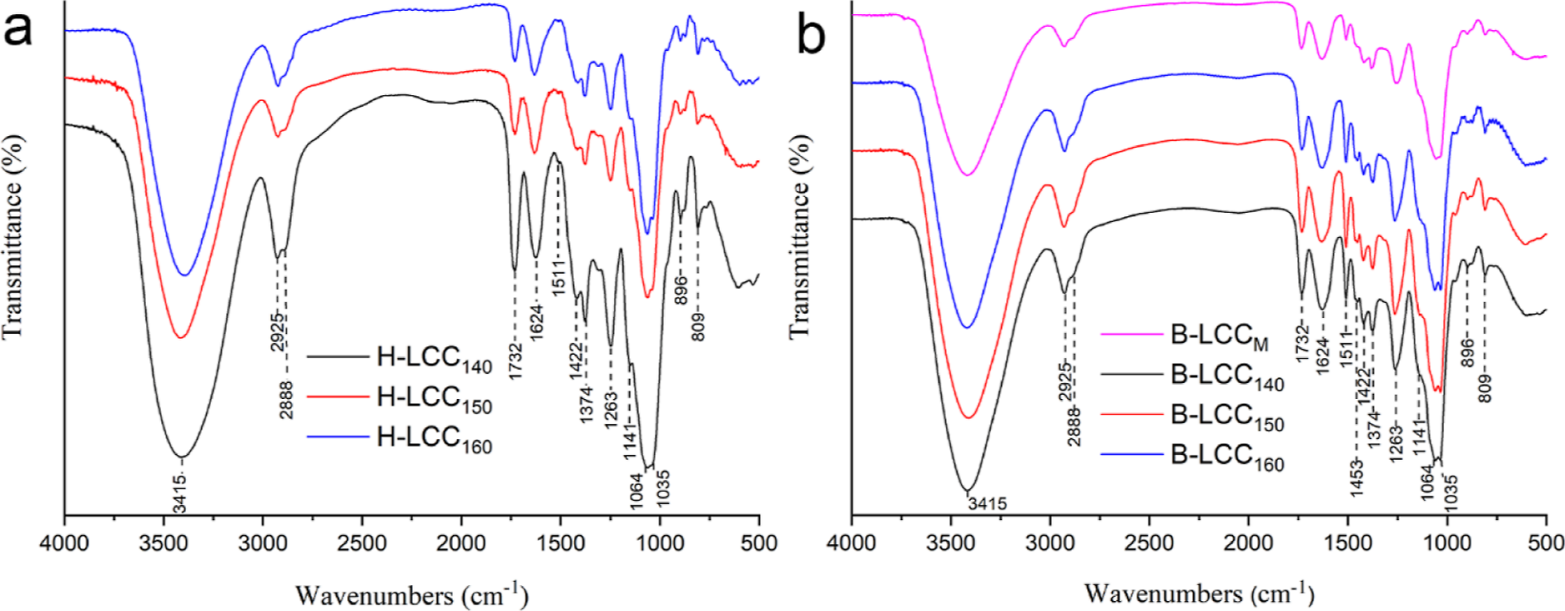
FTIR spectra of H-LCCs (a) and B-LCCs (b) obtained after pre-hydrolysis at 140, 150, and 160 °C for 120 min.

FTIR spectroscopy of H-LCC was conducted to provide further evidence on the chemical transformations taking place during pre-hydrolysis (Figure 3a). Given the similar characteristic bands, it is concluded that the main structures of H-LCCs were similar after pre-hydrolysis at 140, 150, and 160 °C for 120 min.

According to previous studies, the absorbance at around 1732 cm^–1^ is related to the C=O stretch in unconjugated ketone, carbonyl, and ester groups. The large intensity of the band in H-LCCs correlated with the higher content of mannan acetylated at C-2 and C-3. The decrease in the intensity at higher treatment severities was indicative of the deacetylation. The absorptions located at 1624, 1511, and 1422 cm^–1^ were related to the stretching vibration of the aromatic ring, which confirmed the existence of lignin in the LCCs.^3^ The absorptions at 1141 and 1064 cm^–1^ are characteristics of arabinan and xylan, respectively.^28^

The FTIR spectra of B-LCC samples are shown in Figure 3b. Compared with H-LCC, the methoxyl C–H bending and C–C stretching in the aromatic skeleton represented by the peaks at 1453 cm^–1^ indicated a higher lignin content in B-LCCs. Meanwhile, a stronger signal intensity at 1511 cm^–1^ indicated the increase of lignin content in B-LCC.

**Figure 4:**
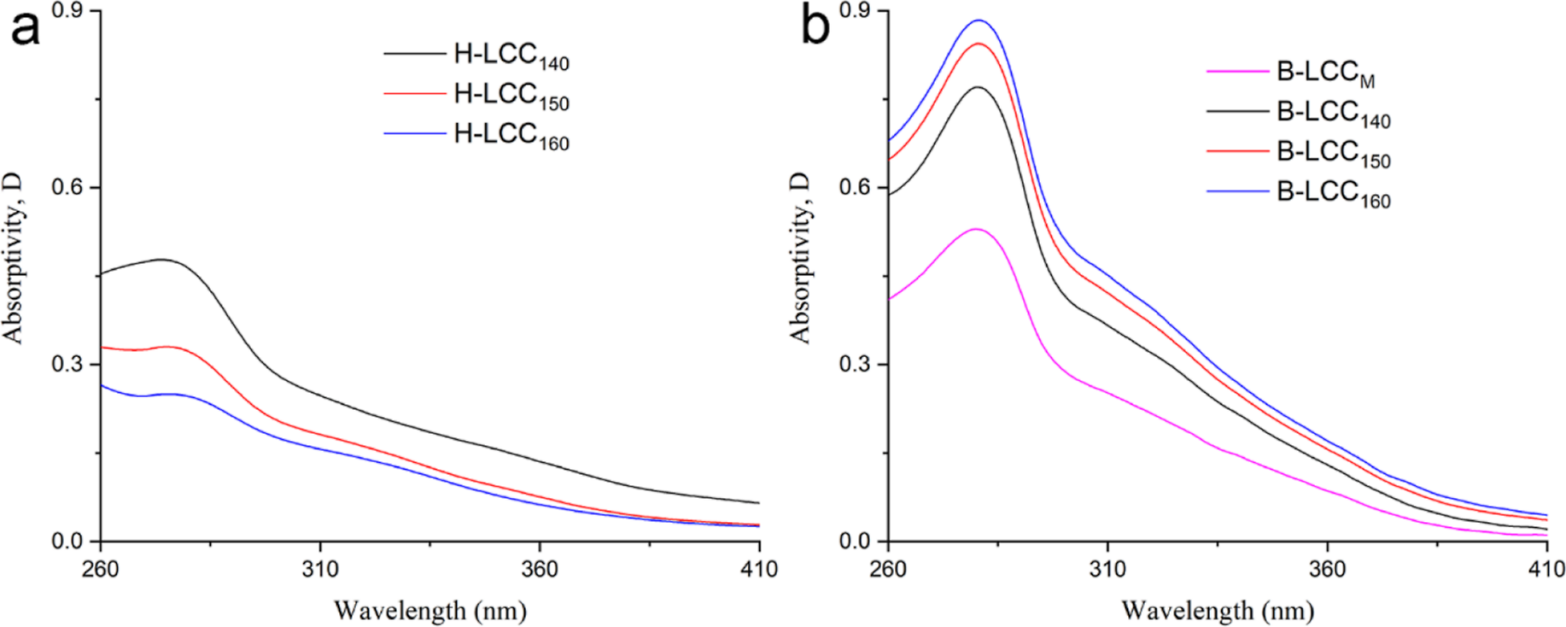
UV–vis spectra of H-LCCs (a) and B-LCCs (b) obtained after pre-hydrolysis at 140, 150, and 160 °C for 120 min. Second-derivative spectra and difference spectra of the LCCs are also listed in Figures S1 and S2, respectively.

The UV–vis spectra of H-LCC samples are shown in Figure 4a. Usually, the spectrum of softwood lignins includes a maximum at 280 nm.^29^ The spectra of H-LCCs exhibited a maximum at 272 nm. The hypochromic changes indicated some reactions leading to the blocking of phenolic hydroxyl groups that might happen during pre-hydrolysis. Second-derivative ultraviolet spectroscopy was used to highlight the fine structure of spectral curves (Fig. S1a). Unfortunately, no significant changes were found among the H-LCC samples. The “difference spectrum” (spectrum that is obtained by subtraction of the absorption curves of two components) of H-LCCs showed a generally higher difference absorbance throughout the whole wavelength range, between 260 and 410 nm (Fig. S2a). Considering the insignificant changes in lignin structure, it can be suggested that the difference noted are mainly caused by the lignin content.

The UV–vis spectra of B-LCCs are shown in Figure 4b. The maximum absorbance is presented at 280 nm, and a weak absorption was found near 320 nm. It has been suggested that unsaturation is introduced into the side chain of the lignin macromolecule during pre-hydrolysis.^30^ Besides, the difference spectra for the B-LCCs (Figure S2b) showed a generally higher difference in absorbance, especially at the beginning of pre-hydrolysis (<140 °C).

### 3.5 LCC Nuclear Magnetic Resonance

2D HSQC NMR spectroscopy is of great potential in the characterization for lignin–carbohydrate complexes and provide valuable information, including lignin structure, carbohydrate composition, and linkages (Table 3). The 2D HSQC NMR spectra of H-LCC and B-LCC samples are shown in Figure S3 and S4, respectively.^31,32^

**Table 3:** Assignments of ^13^C–^1^H Cross-Signals in the 2D HSQC NMR Spectra of H-LCC Samples Obtained by Pre-hydrolysis at 140, 150, and 160 °C for 120 min^a^

Label	δC/δH (ppm)	assignments	H-LCCs			B-LCCs			
			140	150	160	M	140	150	160
Lignin Cross-Signals									
OCH_3_	56.0/3.73	C–H in methoxyls	√	√	√	√	√	√	√
A_β(G)_	83.7/4.28	Cβ–Hβ in β-O-4′ linked to G	√			√	√	√	√
B_α_	85.1/4.61	Cα–Hα in β–β′ resinol				√	√	√	√
B_β_	53.9/3.07	Cβ–Hβ in β–β′ resinol				√	√	√	√
C_α_	86.8/5.43	Cα–Hα in phenylcoumaran				√	√	√	√
C_β_	53.8/3.44	Cβ–Hβ in phenylcoumaran				√	√	√	√
G_2_	110.8/6.96	C_2_–H_2_ in guaiacyl units	√	√	√	√	√	√	√
G_5_	114.9/6.68	C_5_–H_5_ in guaiacyl units				√	√	√	√
G_6_	119.1/6.73	C_6_–H_6_ in guaiacyl units	√	√	√	√	√	√	√
Carbohydrate Cross-Signals									
X_1_	102.2/4.24	C_1_–H_1_ in β-d-xylopyranose	√	√	√	√	√	√	√
X_3_	74.6/3.24	C_3_–H_3_ in β-d-xylopyranose	√	√	√	√	√	√	√
X_4_	75.6/3.56	C_4_–H_4_ in β-d-xylopyranose	√	√	√	√	√	√	√
X_5_	63.7/3.15–3.86	C_5_–H_5_ in β-d-xylopyranose	√	√	√	√	√	√	√
Ara_1_	108.6/4.76	C_1_–H_1_ in α-l-arabinofuranose	√	√					
Ara_2_	82.3/3.80	C_2_–H_2_ in α-l-arabinofuranose	√	√	√	√			
Ara_5_	63.1/3.39–3.69	C_5_–H_5_ in α-l-arabinofuranose	√	√	√	√	√	√	√
Glc_1_	103.3/4.29	C_2_–H_2_ in β-d-glucopyranose	√	√	√	√	√	√	√
Glc_5_	71.5/3.10	C_5_–H_5_ in β-d-glucopyranose	√	√	√	√	√	√	√
Man_6_/Glc_6_	61.6/3.62	C_6_/H_6_ in β-d-glucopyranose/mannopyranose	√	√	√	√	√	√	√
Man_1_	100.9/4.53	C_1_–H_1_ in β-d-mannopyranose	√	√	√	√	√	√	√
Man_2_	74.2/3.08	C_2_–H_2_ in β-d-mannopyranose	√	√	√	√	√	√	√
Man_2_-Ac	71.8/5.21	C_2_–H_2_ in 2-O-acetyl β-d-mannopyranose	√	√	√	√	√	√	√
Man_3_	76.2/3.31	C_3_–H_3_ in β-d-mannopyranose	√	√	√	√	√	√	√
Man_3_-Ac	71.8/4.73	C_3_–H_3_ in 3-O-acetyl β-d-mannopyranose	√			√	√	√	√
Man_4_	79.8/3.35	C_4_–H_4_ in β-d-mannopyranose	√	√	√	√	√	√	√
Man_5_	77.7/3.62	C_5_–H_5_ in β-d-mannopyranose	√	√	√	√	√	√	√
Gal_1_	105.9/4.25	C_1_–H_1_ in β-d-galactopyranose	√	√	√	√	√	√	√
LC Cross-Signals									
Est	63.5/4.23	esters to the lignin γ-OH	√	√	√	√	√	√	√
BE_1_	81.4/4.52	benzyl ether				√	√	√	√
PG	102.3/4.90	phenyl glycoside	√	√	√				

a The changes in chemical structure at different temperatures during pre-hydrolysis were identified. The information about B-LCC is also listed to further illustrate the changes. The 2D HSQC NMR spectra of H-LCC and B-LCC are included in Figures S3 and S4, respectively. √ means that the structure is confirmed in LCCs.

#### 3.5.1 Lignin Structure

δ_C_/δ_H_ 56.0/3.73 corresponded to methoxy groups (OCH_3_). The C_β_-H_β_ cross-peak of the β-O-4′ structure linked to the guaiacyl unit was observed at δ_C_/δ_H_ 83.7/4.28 (*A*_β(*G*)_). The C_2_–H_2_ and C_6_–H_6_ cross-peak in guaiacyl units was found at δ_C_/δ_H_ 110.8/6.96 (*G*_2_) and 119.1/6.73 (*G*_6_), respectively. Compared with data for B-LCC, β–β′ resinol (B) and phenylcoumaran (C) cross-peaks were not shown in the side chain region of H-LCC. As the reaction temperature increased, the cleavage of lignin aliphatic C–O linkages occurred. Only a small number of guaiac-based units linked to β-O-4′ existed in H-LCC_140_. This reflects the fact that the dissolved lignins were extensively degraded under such conditions. Also, it is noted that the C_5_–H_5_ cross-peak in the guaiacyl unit was not found. This indicates that a certain condensation occurred at the C5 position of the lignin’s benzene ring.

#### 3.5.2 Carbohydrate Composition

The cross-peaks at δ_C_/δ_H_ 102.2/4.24 (H_1_/C_1_), 74.6/3.24 (H_3_/C_3_), 75.6/3.56 (H_4_/C_4_), and 63.7/(3.15; 3.86) (H_5_/C_5_) were from β-d-xylopyranose, and those at δ_C_/δ_H_ 108.6/4.76 (H_1_/C_1_), 82.3/3.80 (H_2_/C_2_), and 63.1/(3.39; 3.69) (H_5_/C_5_) were from α-l-arabinofuranose. The results indicated that AGX existed in both H-LCCs and B-LCCs. The cross-peaks at δ_C_/δ_H_ 103.3/4.29 (H_1_/C_1_) and 71.5/3.10 (H_5_/C_5_) were from β-d-glucopyranose. In addition, the cross-peaks at δ_C_/δ_H_ 100.9/4.53 (H_1_/C_1_), 74.2/3.08 (H_2_/C_2_), 76.2/3.31 (H_3_/C_3_), 79.8/3.35 (H_4_/C_4_), 77.7/3.62 (H_5_/C_5_), and 61.6/3.62 (H_6_/C_6_) were from β-d-mannopyranose. The results confirmed that GGM was also present in the LCCs. In addition, the acetylated mannose in H-LCCs gradually disappeared at elevated temperatures. That was because acetic acid could release from the acetylated polysaccharides and further lowered the pH of the extract to allow the hydrolysis of glycosidic linkages in hemicellulose and β-ether linkages in lignin.^33^

#### 3.5.3 Lignin–Carbohydrate Linkages

The predominant types of native LCC are phenyl glycosides (PG), benzyl ethers (BE), and γ-esters (Est).^13^ Phenyl glycoside linkages gave a group of carbohydrate signals C-1 at 104–102.3/4.90 ppm. γ-Esters with C_γ_/H_γ_ were subjected to resonance at 63.5/4.23 ppm. Phenyl glycoside (PG) and esters to the lignin γ-OH (Est) linkages were both determined in H-LCC. Benzyl ethers referred to linkages between the α-position of lignins and OH groups of carbohydrates. The cross-peaks from BE_1_ were found at 81.4/4.52 only in the HSQC spectra of B-LCCs.

#### 3.5.4 LCC and Lignin Substructure Semiquantification

2D HSQC and ^13^C NMR were employed to semiquantify LCC and lignin substructures.^19,20^ The results of the linkage semiquantification in the preparations are summarized in Table 4. As for lignin linkages, the changes of B-LCC on pre-hydrolysis temperature included a decrease in aryl ether (β-Ο-4′) content and some increase in resinol (β–β′) and phenyl coumaran (β-5′) content. The number of β-O-4′ linkages decreased from 71.8/100Ar in the material to 47.2/100Ar in B-LCC_160_. The content of β-5′ and β–β′ structures in B-LCC increased. The β-O-4′ linkages in lignocellulose were easily activated and cleaved during pre-hydrolysis. Meanwhile, only 1.1/100Ar of β-O-4′ linkages in H-LCCs were more noteworthy. The possibility of condensation reaction at the C5 position of lignin’s benzene ring can be proposed. At present, lignin models are being used to identify the condensation reaction of lignins during pre-hydrolysis.

**Table 4:** Semiquantification of LCC and Lignin Substructures from the Spectra Acquired on a 600 MHz Spectrometer^a^

preparations	amounts, per 100 Ar					
	β-O-4′	β–β′	β-5′	Est	BE	PG
H-LCC_140_	1.1	N.D.	N.D.	2.1	N.D.	11.9
H-LCC_150_	N.D.	N.D.	N.D.	4.5	N.D.	6.7
H-LCC_160_	N.D.	N.D.	N.D.	4.7	N.D.	3.3
B-LCC_M_	71.8	4.6	13.2	16.3	18.3	N.D.
B-LCC_140_	68.6	4.9	14.4	15.5	17.9	N.D.
B-LCC_150_	58.8	5.4	17.6	14.7	17.4	N.D.
B-LCC_160_	47.2	5.9	19.6	10.9	15.9	N.D.

a N.D indicates “not detected”. The ^13^C NMR spectra of H-LCCs and B-LCCs are shown in Figures S5 and S6, respectively.

As for lignin-carbohydrate linkages, though the BE linkage was much more stable in lignocellulosic biomass, the content in B-LCC still decreased with the hydrolysis temperature.^34^ PG linkage decomposed more easily during pre-hydrolysis. We found that the amount of PG linkages in H-LCC was greatly reduced, from 11.9/100 Ar at 140 °C to 3.3/100 Ar at 160 °C. Just like the PG linkage, ester-linkages are also pH-labile and would rapidly hydrolyze in acidic solution.^35^ It is interesting that the Est content in H-LCC increased with the hydrolysis temperature. However, there was no strong evidence for this increase. Acetyl transfer also occurred from hemicellulose to lignin during pre-hydrolysis.^14^ It is known that the traditional NMR signal for ester linkages is difficult to detect and overlaps with the specific ester.^36^ Further evidence from lignin model compounds is needed to obtain more insights on the lignin–carbohydrate linkages.

## 4 Conclusions

We reveal the composition and structure of LCCs present in pre-hydrolysis liquors. C–C lignin bonds (β–β′ and β-5′) were relatively stable during pre-hydrolysis and only 8.2% of lignins were degraded via β-O-4′ linkage breaking. Glucomannan, a major hemicellulose, was completely removed and was associated with the degraded lignin fragments via ether and ester bonds. Meanwhile, the newly formed LCCs were not stable at high-temperature and acid conditions. These findings are relevant to the enhancement of the separation and application of LCCs obtained from lignocellulosic biomass during the pre-hydrolysis processes.

## Supporting Information Available

Absolute mass of the chemical compositions; degradation of chemical compositions during the pre-hydrolysis process; absolute mass of the chemical compositions; second-derivative spectra of H-LCCs and B-LCCs; difference spectra of H-LCCs and B-LCCs; HSQC spectra of the H-LCCs; HSQC spectra of the B-LCCs; ^13^C NMR spectra of H-LCCs; and ^13^C NMR spectra of B-LCCs (PDF)
