# Supplementary material for: Formation and Identification of Lignin–Carbohydrate Complexes in Pre-hydrolysis Liquors
Source: Biomacromolecules. 2023 Jun 2;24(6):2541–8. doi: 10.1021/acs.biomac.3c00053 (PMC10265660; doi:10.1021/acs.biomac.3c00053)
Supplement: Supplementary file 1 — bm3c00053_si_001.pdf [file bm3c00053_si_001.pdf]

Supplementary material

Formation and identification of lignin-carbohydrate complexes in pre-hydrolysis liquors

Nianjie Feng[a,b,1], Shaowen She[a,1], Fei Tang[a], Xiangdong Zhao[a], Jingqian Chen[b], Peng Wang[a], Qian Wu[a,b,], Orlando J. Rojas[b,c,*]*

[a] Key Laboratory of Fermentation Engineering (Ministry of Education), Hubei Key Laboratory of Industrial Microbiology, National "111" Center for Cellular Regulation and Molecular Pharmaceutics, Hubei Research Center of Food Fermentation Engineering and Technology, Hubei University of Technology, Wuhan 430068, China

[b] Bioproducts Institute, Department of Chemical & Biological Engineering, Department of Chemistry, and Department of Wood Science, 2360 East Mall, The University of British Columbia, Vancouver, BC V6T 1Z3, Canada.

[c] Department of Bioproducts and Biosystems, School of Chemical Engineering, Aalto University, Vuorimiehentie 1, FI-00076, Espoo, Finland

[*]Corresponding author: Orlando J. Rojas, E-mail address: orlando.rojas@ubc.ca; Tel: +1 604 822 3457

[1]Indicates equal contribution.

Table S1 The absolute mass of the chemical compositions. The data were based on 40 g (oven dry) raw material used in the pre-hydrolysis. N.D indicates "not detected".

Chemical compositions	Raw material	Residues			Extracts		
		140	150	160	140	150	160
Lignin (g)	11.9	11.4	11.2	11.0	0.2	0.3	0.4
Glucose (g)	17.5	16.2	15.9	15.4	0.5	0.7	0.9
Xylose (g)	2.1	0.8	0.7	0.6	0.5	0.6	0.6
Galactose (g)	1.2	0.3	0.2	0.2	0.3	0.4	0.4
Arabinose (g)	0.3	0.0	N.D	N.D	0.1	0.1	0.1
Mannose (g)	6.5	2.1	1.9	1.2	1.7	1.8	2.1
4-O-methyl-glucuronic acid (g)	0.6	0.5	0.5	0.5	0.1	0.1	0.1

Table S2 The degradation of chemical compositions during pre-hydrolysis process. N.D indicates "not detected".

Chemical compositions	Degradation (%)		
	140	150	160
Lignin	4.7	5.7	7.8
Glucose	7.4	9.2	12.0
Xylose	62.6	66.7	71.1
Galactose	70.3	79.7	86.1
Arabinose	87.7	N.D	N.D
Mannose	67.0	71.4	81.6
4-O-methyl-glucuronic acid	7.8	9.9	13.6

Table S3 The absolute mass of the chemical compositions. The data were based on 40 g (oven dry) raw material used in pre-hydrolysis. Low indicates "mass less than 0.01 g".

Chemical compositions	H-LCCs			B-LCCs			
	140	150	160	M	140	150	160
Yield (g)	0.35	0.76	0.90	5.40	2.79	1.65	0.74
Lignin (g)	0.02	0.04	0.05	1.60	1.11	0.77	0.38
Glucose (g)	0.04	0.16	0.21	0.69	0.38	0.22	0.10
Xylose (g)	0.04	0.09	0.09	0.41	0.19	0.08	0.02
Galactose (g)	0.05	0.13	0.13	1.07	0.28	0.13	0.06
Arabinose (g)	Low	Low	Low	0.04	0.01	Low	Low
Mannose (g)	0.10	0.32	0.41	1.52	0.78	0.42	0.17
4-O-methyl-glucuronic acid (g)	Low	0.01	0.02	0.08	0.05	0.03	0.01

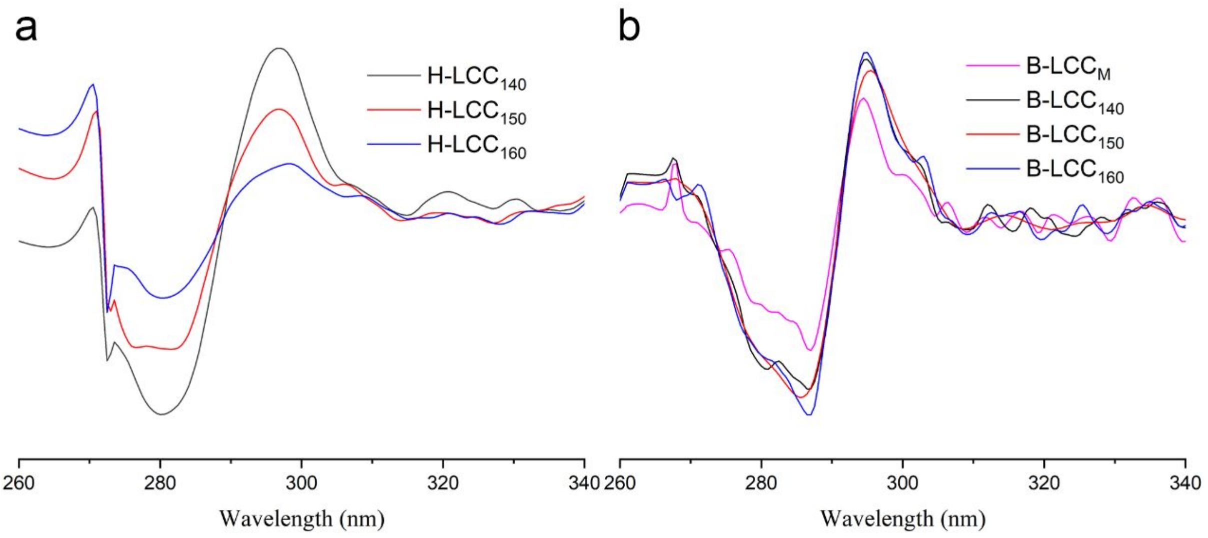

Figure S1 The second derivative spectra of H-LCCs (a) and B-LCCs (b).

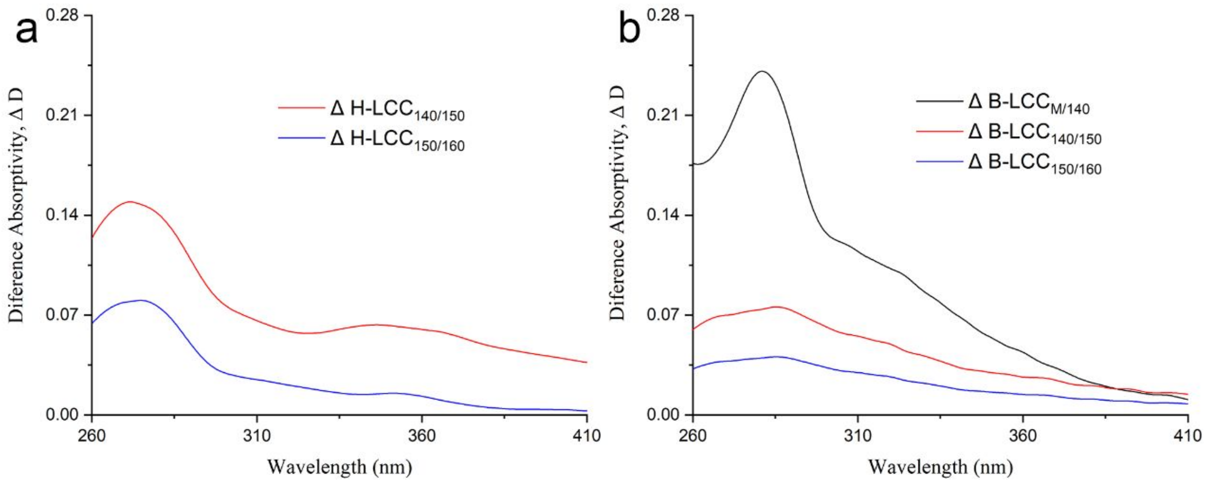

Figure S2 The difference spectra of H-LCCs (a) and B-LCCs (b).

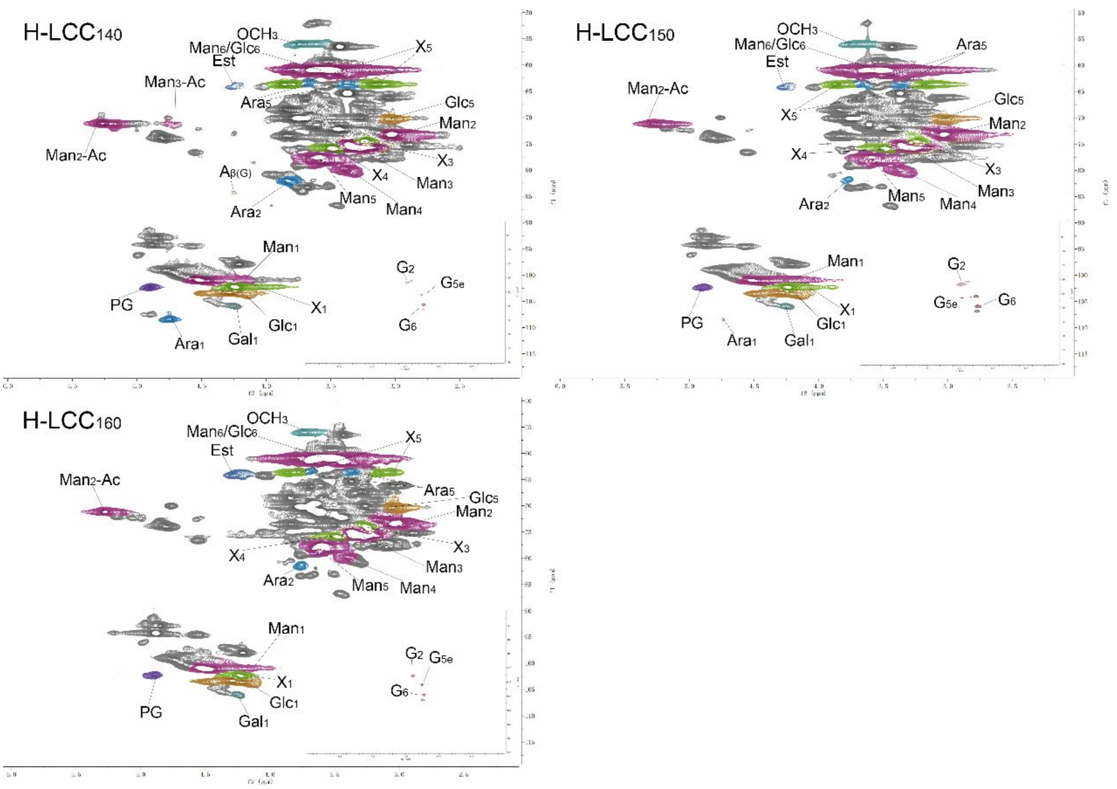

Figure S3 Aromatic regions (δC/δH 140–90/8.5–5.5) and aliphatic regions (δC/δH 120–50/6.0–2.0) in HSQC spectra of the H-LCCs. (A) β-O-4′ alkyl-aryl ether; (G) guaiacyl unit; (X) β-D-xylopyranose; (Ara) α-L-arabinofuranose; (Glc) β-D-glucopyranose; (Man) β-D-mannopyranose; (Gal) β-D-galactopyranose; (Est) esters to the lignin γ-OH; (PG) phenyl glycoside.

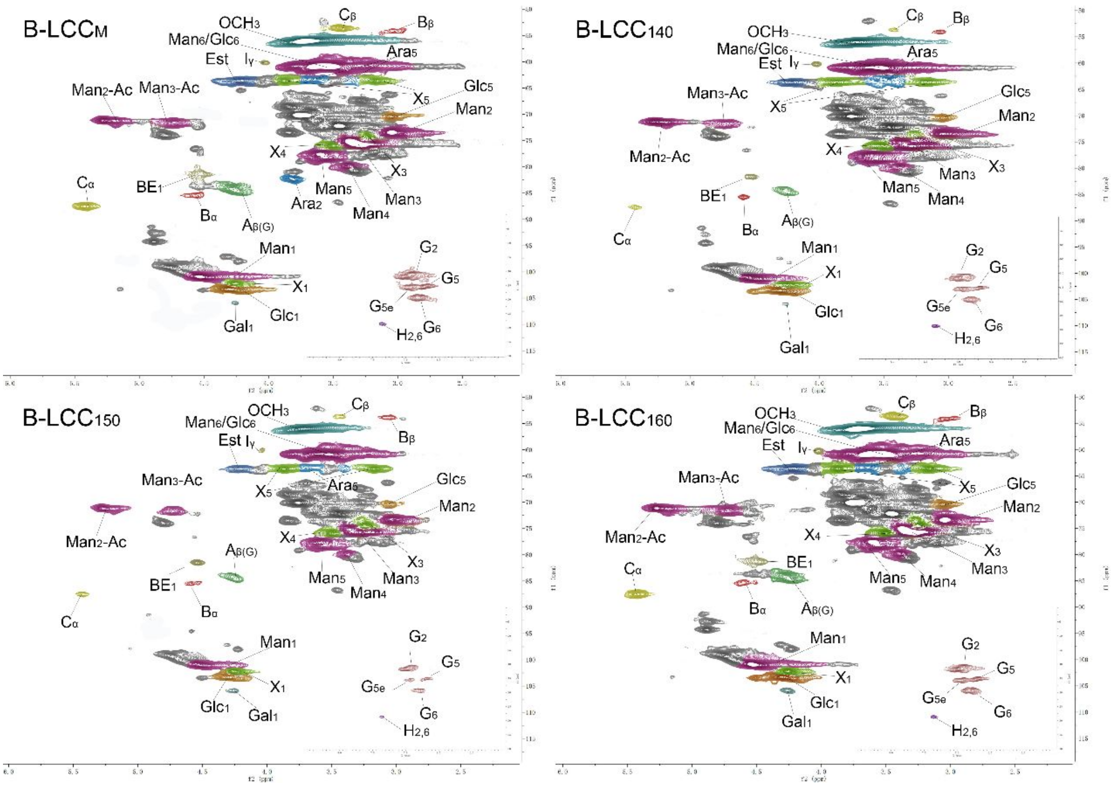

Figure S4 Aromatic regions (δC/δH 140–90/8.5–5.5) and aliphatic regions (δC/δH 120–50/6.0–2.0) in HSQC spectra of the B-LCCs. (A) β-O-4´ alkyl-aryl ether; (B) β-β´ resinol; (C) phenylcoumaran; (G) guaiacyl unit; (X) β-D-xylopyranose; (Ara) α-L-arabinofuranose; (Glc) β-D-glucopyranose; (Man) β-D-mannopyranose; (Gal) β-D-galactopyranose; (Est) esters to the lignin γ-OH; (BE) benzyl ether.

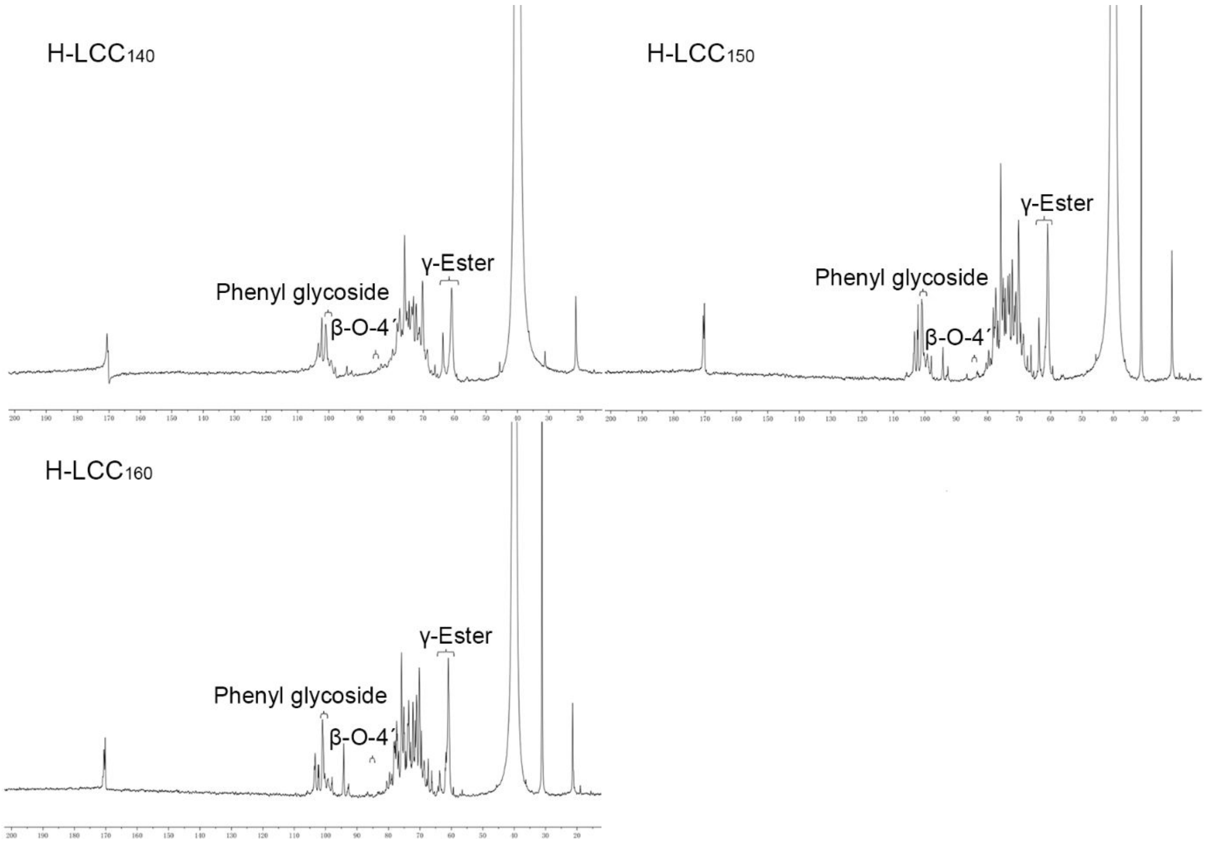

Figure S5 Quantitative ^{13}C NMR spectra of H-LCCs. β-O-4′ (85.2-83.5ppm); phenyl glycoside (102- 99 ppm); γ-ester (65- 58 ppm).

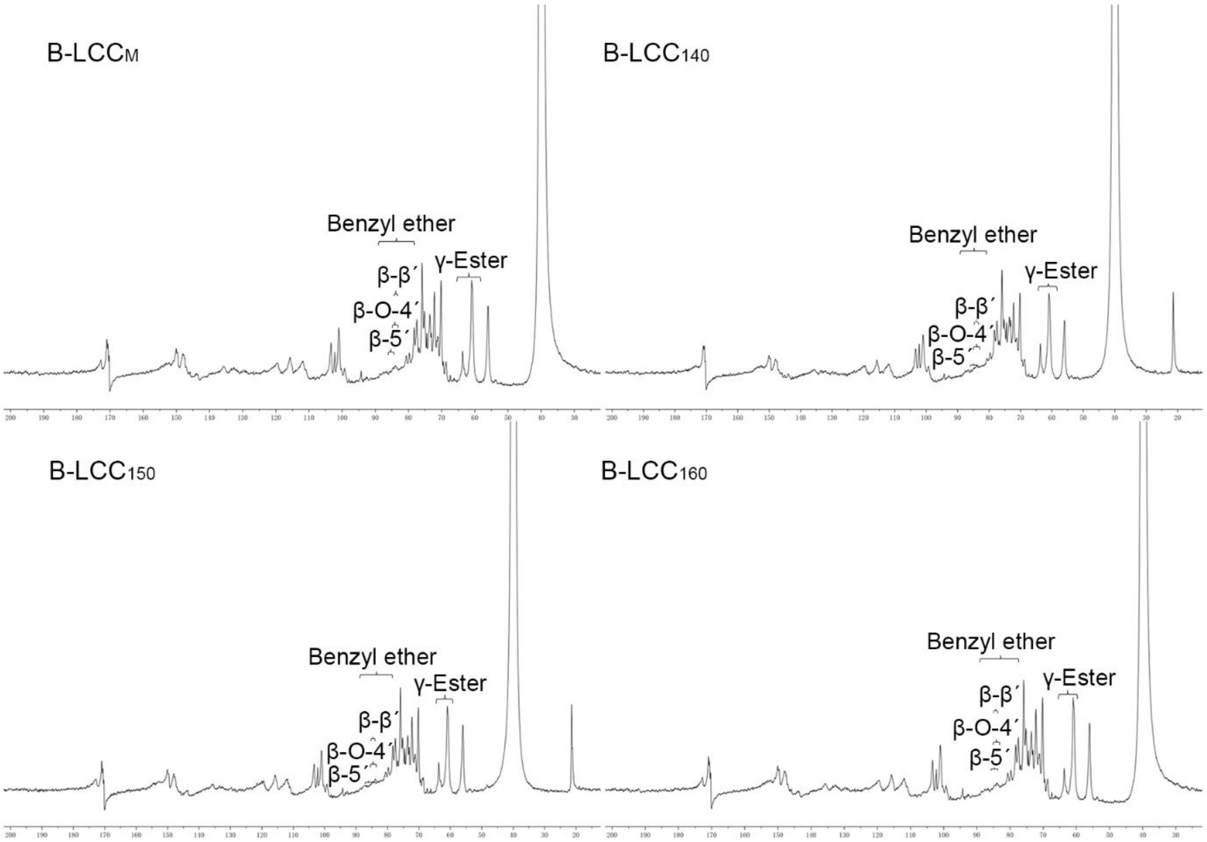

Figure S6 Quantitative ^{13}C NMR spectra of B-LCCs. β-O-4´ (85.2- 83.5 ppm); β-β´ (85.1- 84.4 ppm); β-5´ (86.8- 84.7 ppm); benzyl ether (90- 78 ppm); γ-ester (65- 58 ppm).
